# A Case of Granulomatosis with Polyangiitis with Various Breast Lesions as the Initial Symptoms: A Case-Based Review

**DOI:** 10.1155/2021/4416072

**Published:** 2021-08-20

**Authors:** Masatoshi Kawataka, Toshiki Kido, Reina Tsuda, Takafumi Onose, Ryoko Asano, Miho Yamazaki, Naonori Sugishita, Hiroyuki Hounoki, Toshiko Kakiuchi, Koichiro Shinoda, Kazuyuki Tobe

**Affiliations:** ^1^First Department of Internal Medicine, University of Toyama, Toyama, Japan; ^2^Department of Diagnostic Pathology, University of Toyama, Toyama, Japan

## Abstract

A 44-year-old woman presenting with pus-like discharge from the nipples visited our hospital for scleritis. Subcutaneous induration and ulceration were found on her breast. She was diagnosed with granulomatosis with polyangiitis (GPA) considering scleritis, sinusitis, cutaneous granuloma formation, and antiproteinase 3-antineutrophil cytoplasmic antibodies and was successfully treated with glucocorticoids. Fifteen months later, she developed pulmonary consolidation and a right breast nodule. Biopsies of the breast nodule showed granulomatous vasculitis, and she was treated with rituximab. While breast involvement in GPA is rare, unilateral breast mass is a typical clinical feature; thus, GPA should be considered in such cases.

## 1. Introduction

Granulomatosis with polyangiitis (GPA) is a systemic necrotizing vasculitis associated with the presence of antiproteinase 3-antineutrophil cytoplasmic antibodies (PR3-ANCA). The most common sites of organ involvement are the upper and lower respiratory tracts and kidneys, and clinical manifestations are granulomatous and necrotizing inflammatory lesions and rapidly progressive pauci-immune glomerulonephritis [1]. By contrast, breast involvement in GPA is rare [2–4]; as far as we could find in the English literature, only one case has been reported in Japan [5]. A unilateral breast tumor-like lesion is a typical clinical and imaging feature and is sometimes misdiagnosed as breast cancer. We, herein, report the case of a patient with GPA who presented with bilateral breast lesions as initial symptoms.

## 2. Case Presentation

**Figure 1:**
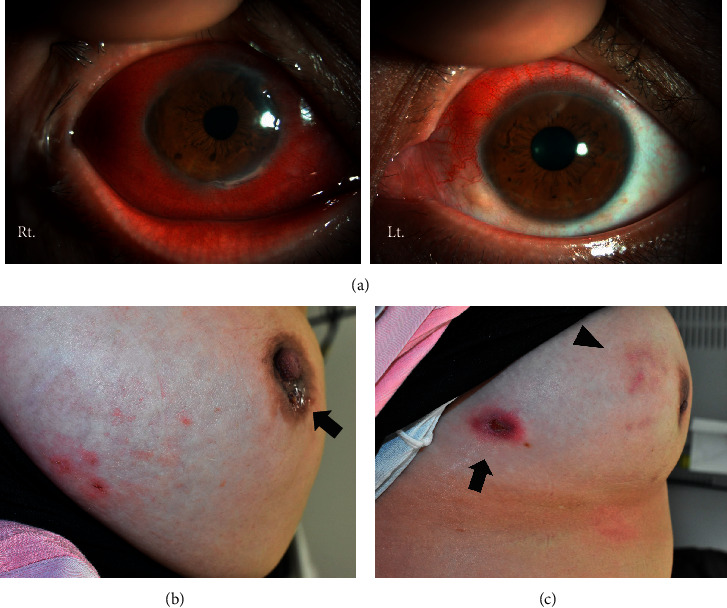
(a) Clinical pictures of the patient's bilateral necrotizing scleritis. (b) Discharge from her nipples (arrow). (c) Pale erythema (arrowhead) and a skin ulcer (arrow) on the right breast.

**Figure 2:**
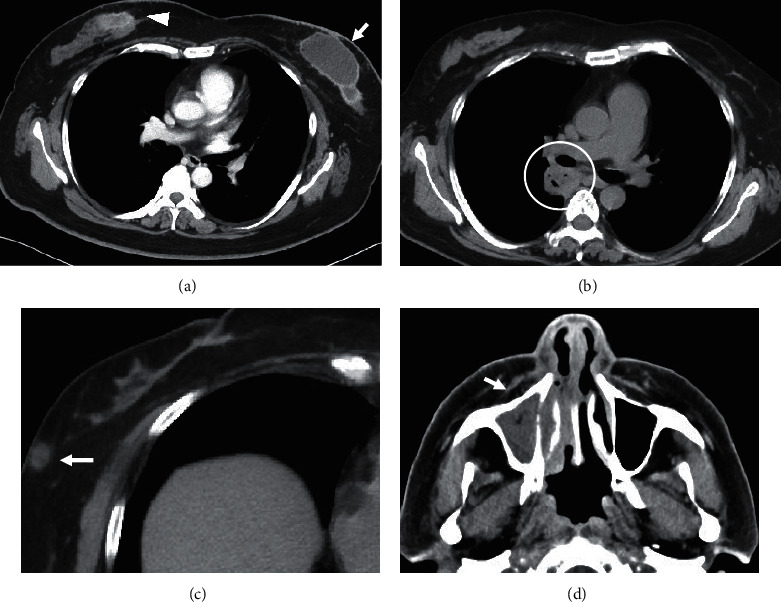
(a) Computed tomography showing tumor-like lesions in the patient's breasts (right, arrowhead; left, arrow) on the first admission. (b) Whole-body computed tomography revealing pulmonary consolidation in the right lower lobe (circle). (c) A small nodule in the right breast (arrow), and (d) recurrence of right maxillary sinusitis (arrow) during the second admission.

**Figure 3:**
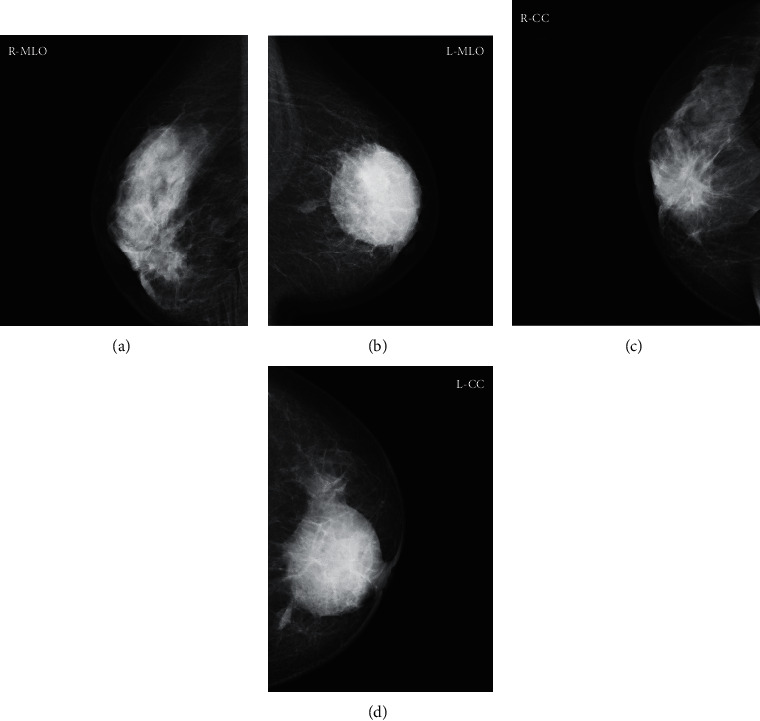
Mediolateral oblique (a, b) and craniocaudal (c, d) mammography demonstrates a round, microlobulated, and high-density mass lesion in the right breast (a, c) and circumscribed, round, and high-density mass lesion in the left breast (b, d).

A 44-year-old woman visited the department of ophthalmology in our hospital because of hyperemia in both eyes, which had rapidly worsened two months prior. She was diagnosed with bilateral necrotizing scleritis (Figure 1(a)) and was referred to our department to assess the involvement of an autoimmune disease. Her medical history revealed that she had been experiencing pus-like discharge from bilateral nipples for the past three months and had thus visited a dermatologist; she was treated with antibiotics, but there was no improvement. More recently, she had suffered from nasal congestion and discharge. She was afebrile, and auscultation revealed no rales in her lung fields and no abnormal heart sounds. Subcutaneous induration was observed in both breasts, along with discharge from the nipples (Figure 1(b)). Pale erythema and a skin ulcer were found on the right breast (Figure 1(c)). No lymph node enlargement was observed from the neck to the axilla. Laboratory test results showed an elevated white blood cell count (10,730/*μ*L) with high polymorphonuclear neutrophil level (70.1%), normal liver and renal function, C-reactive protein (CRP) in almost normal range (1.2 mg/L), and a slightly elevated erythrocyte sedimentation rate (ESR) (48 mm/h). Immunological tests were negative for rheumatoid factor, antinuclear antibodies, and myeloperoxidase- (MPO-) ANCA; however, PR3-ANCA was slightly elevated (5.9 U/mL; normal value <2.0 U/mL). The patient tested negative for tuberculosis (TB) in an interferon-*γ* releasing assay. Urinalysis showed no abnormalities, including hematuria and proteinuria. Computed tomography (CT) of the thorax indicated no abnormalities in the lung fields; however, tumor-like lesions were found in her breasts (Figure 2(a)). CT of the paranasal sinuses showed sinusitis in the right maxillary sinus, and nasopharyngolaryngoscopy demonstrated purulent discharge from the middle meatus. Mammography demonstrated a round, microlobulated, and high-density mass lesion in the right breast, which was classified as category 4, and a 7.5 cm circumscribed, round, and high-density mass lesion in the left breast, which was classified as category 3 (Figure 3). Ultrasonography demonstrated a well-defined, rough-margined, heterogeneous, low-echoic, and unmeasurable giant mass lesion in the left breast and an irregular-shaped, ill-defined, heterogeneous mass lesion in the right breast. Breast cancer, fibroadenoma, sclerosing adenosis, mastitis, and abscess formation were differential diagnoses.

**Figure 4:**
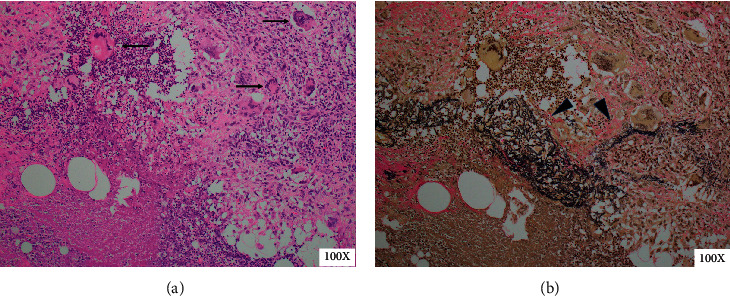
Histopathological findings of the biopsy of the nodule in the right breast. Lymphocyte-dominant inflammatory cell infiltration and granulomas with polynuclear giant cells (arrow) are visible around the blood vessels in the subcutaneous fat layer with hematoxylin and eosin staining (a). Verhoeff's Van Gieson staining shows destruction of the elastic plate (b) (arrowheads).

A bacterial culture test and cytological examination of the exudate from the nipples were performed to differentiate between infection and neoplastic lesions. Although a small amount of *Serratia marcescens*, *Corynebacterium* specie*s*, *Anaerococcus* species, *Finegoldia magna*, and *Peptoniphilus* species were present in the bacterial culture, infectious mastitis was ruled out considering her clinical course. Cytology revealed infiltration of inflammatory cells, which were mainly lymphocytes and agglomeration of epithelioid cells containing multinucleated giant cells, without malignant cells. Core needle biopsy of her left breast (from the location of the arrow in Figure 2(a)) showed inflammatory cell infiltration consisting of lymphocytes with surrounding fibrotic lesions without vasculitis. A biopsy of a skin ulcer on the right chest wall (from the location of the arrow in Figure 1(c)) showed granuloma formation, including lymphocytes and plasma cells outside the blood vessels without vasculitis. These lesions were suspected as granulomatous mastitis considering breast imaging, nonsignificant bacterial culture, and cytological and pathological examination. Based on the presence of bilateral scleritis, cutaneous granuloma formation, inflammatory breast masses, right maxillary sinusitis, and PR3-ANCA positivity, the patient was diagnosed with a probable case of localized GPA according to the diagnostic criteria of the Ministry of Health, Labor and Welfare of Japan. She was treated with methylprednisolone pulse therapy, followed by a daily dose of 40 mg of prednisolone (PSL) and trimethoprim/sulfamethoxazole. Her symptoms improved, and the titers of CRP, ESR, and PR3-ANCA were normalized. Fifteen months later, when PSL was tapered to 5 mg/day, she complained of cough and nasal discharge. The titer of CRP and ESR had increased to 95.8 mg/L and 80 mm/h. The PR3-ANCA titer had increased to 26.5 U/mL, and whole-body CT revealed pulmonary consolidation in the right lower lobe (Figure 2(b)) and a small nodule in the right breast (Figure 2(c)) as new lesions with recurrence of maxillary sinusitis (Figure 2(d)). A biopsy of the small nodule in the right breast (from the location of the arrow in Figure 2(c)) showed lymphocyte-dominant inflammatory cell infiltration and granulomas with polynuclear giant cells around the blood vessels in the subcutaneous fat layer, which can be interpreted as necrotizing granulomatous vasculitis (Figures 4(a) and 4(b)). Granulomatous lesions were found in the nasal cavity, and a biopsy of this site showed necrotizing granulomatous inflammation with giant cells. These lesions did not contain caseous necrosis. Thus, the diagnosis was conclusively confirmed as a case of localized GPA; daily PSL was increased to 10 mg, and remission induction therapy for GPA, consisting of four once-weekly doses of 375 mg/m^2^ rituximab (RTX), was introduced. After treatment, all symptoms gradually resolved, and the titers of CRP, ESR, and PR3-ANCA normalized.

## 3. Discussion

Considering the overall clinical course and the positive PR3-ANCA titer, this case was confirmed as a localized form of GPA. However, the initial symptom was pus-like discharge from the nipples that had developed three months prior to hospitalization. Breast lesions in GPA are extremely rare, with 35 cases (clinical and pathological features summarized in Table 1) reported to date since the first report by Elsner in 1969 [4–31]. In brief, the female sex was dominant (33 cases), and only two male patients have been reported with GPA complicated by breast lesions [15]. The age ranged from 28 to 81 years, and the average age was 49 years. Ten cases were either positive for cytoplasmic-ANCA or PR3-ANCA, four cases were negative for ANCA, and no cases were positive for MPO-ANCA. There were 28 cases of unilateral breast lesions, 18 of which were predominant on the right side. Seven cases had bilateral breast lesions, as in our case. Breast lesions were initial symptoms and associated with systemic GPA-related lesions in 19 cases, and in 15 cases, breast lesions developed after GPA diagnosis. Breast lesions were characterized by nodule and mass formation in 31 cases, and only one case had discharge from the nipple [6]; there were also six cases of ulceration. In previous reports, most patients utilized glucocorticoid (28 cases) and/or cyclophosphamide (16 cases) treatment. Four patients reported using RTX, and in all cases, remission was achieved with RTX [26–28, 32]. In our case, sinusitis and pulmonary lesions rapidly improved after RTX administration, and there were no breast lesion recurrences.

Ren et al. reviewed breast lesions in systemic vasculitis and described the characteristics of mammographic and ultrasound findings [2]. Accordingly, mammography revealed increased dense mammary parenchyma, heterogeneous breast tissue, vascular calcification, skin or trabecular thickening, and nodular masses. Meanwhile, breast ultrasonography revealed skin thickening, lesions with or without necrosis or calcifications, hypoechoic mammary parenchyma or infiltration of the breast, and penetration of the blood vessels. However, no radiological and ultrasonographical findings characteristic of vasculitis were described [2]. In our case, although breast cancer, fibroadenoma, sclerosing adenosis, mastitis, and abscess formation were differential diagnoses, a pathological examination was necessary for a definitive diagnosis. In our literature review, of the 34 cases with pathological findings, 25 had necrotizing granulomatous vasculitis that could be definitively diagnosed as GPA, and other pathological findings included inflammatory changes with giant cells, panniculitis, and granulomatous mastitis [4]. According to Allende et al., the pathological diagnosis of breast lesions is challenging because small biopsy materials obtained by core needle biopsy made it difficult to conclusively diagnose vasculitis in breast lesions [4]. Accordingly, the initial core needle biopsy did not reveal vasculitis, but nonspecific inflammatory tissue in our case and cytological examination of the exudate from the nipples revealed an increase in neutrophils, histiocytes, multinucleated giant cells, and epithelial cells, which were evaluated as granulomatous mastitis. In cases where the pathological examination of the breast lesion does not provide a definitive diagnosis, GPA is diagnosed from pathological examination of other tissues (e.g., nasal cavity, kidney, and lung). In the present case, we were able to obtain a definitive pathological diagnosis by excisional biopsy of the subcutaneous nodule in the breast and nasal mucosal biopsy at recurrence. Thus, it is important to biopsy multiple organs during pathological diagnosis of GPA.

Our case was the second reported case of breast involvement with GPA in Japan since Oimomi et al. reported the first case in 1980 [5]. The low number of PR3-ANCA-positive GPA cases in Japan [33, 34] may, therefore, attribute to the subsequent low number of GPA cases associated with breast lesions.

Our case was characterized by various breast lesions: the first was a large bilateral breast mass revealed by mammography, the second was purulent discharges from the bilateral nipples (rarely reported in the literature), the third was a skin ulcer that was not continuous with the breast mass, and the fourth was a necrotizing granulomatous vasculitis appearing as a subcutaneous nodule in the breast at recurrence. Although the mechanism of breast lesion development in GPA is unknown, infection in the mammary gland, which is connected to the outside through the nipple, may become the trigger of onset, especially because the infection is involved in the development of ANCA-associated vasculitis [35]. Additionally, *Corynebacterium* species, which might be associated with ANCA formation and disease relapse in GPA [36, 37], have been cultured from the nipple discharges in our case. In this case, not only was the mammary gland affected but the skin and subcutaneous fat tissue—which are not continuous with the mammary gland—were also affected. It is unclear why skin lesions are not systemic and, instead, localized on the breast. However, considering that cutaneous vasculitis is more common in the lower extremities, where blood stasis is more likely, and that local pressure is one of the causes of vasculitis, it is possible that the pressure from women's underwear and bras may trigger vasculitis in the skin and subcutaneous areas of the breast in susceptible patients [38].

In conclusion, breast lesions in GPA are extremely rare, and they may present as the first symptom of the disease or of conditions without the clinical symptoms typical of GPA. If unexplained breast masses, ulceration, or other findings such as discharge from the nipples are encountered, GPA should be included in the differential diagnosis.

## Figures and Tables

**Table 1:** Clinical characteristics of 35 cases of breast lesions with GPA.

Mean age ± SD years	49 ± 14 (28–81)

Gender	Male: 2, female: 33

ANCA	PR3(C)-ANCA: 10
	Negative: 4
	Not described: 21

Location of breast lesion	Bilateral: 7
	Unilateral: 28 (right: 18, left: 9, unknown: 1)

Formation of breast lesion	Nodule/tumor: 31
	Ulceration: 6
	Discharge from nipples: 1

Onset of breast lesion	Initial symptom: 19
	Onset after GPA diagnosis: 15

Pathology of breast lesion	Necrotizing granulomatous vasculitis: 25
	Granulomatous mastitis: 4
	Others (inflammation with giant cells, panniculitis): 5

Treatment	Corticosteroid: 28
	Cyclophosphamide: 16
	Rituximab: 4

## Data Availability

The datasets collected and/or analyzed during the current study are available from the corresponding author on reasonable request.
